# ParcEMon: IoT Platform for Real-Time Parcel Level Last-Mile Delivery Greenhouse Gas Emissions Reporting and Management

**DOI:** 10.3390/s22197380

**Published:** 2022-09-28

**Authors:** Ali Yavari, Hamid Bagha, Harindu Korala, Irfan Mirza, Hussein Dia, Paul Scifleet, Jason Sargent, Mahnaz Shafiei

**Affiliations:** 1 School of Science, Computing and Engineering Technologies, Swinburne University of Technology, Melbourne, VIC 3122, Australia; 2 Institute of Railway Technology, Monash University, Melbourne, VIC 3800, Australia; 3 Department of Civil and Construction Engineering, Swinburne University of Technology, Melbourne, VIC 3122, Australia; 4 School of Business, Law and Entrepreneurship, Swinburne University of Technology, Melbourne, VIC 3122, Australia

**Keywords:** IoT, greenhouse gas, sustainable logistics, last-mile emission, supply chain

## Abstract

Transport is Australia’s third-largest source of greenhouse gases accounting for around 17% of emissions. In recent times, and particularly as a result of the global pandemic, the rapid growth within the e-commerce sector has contributed to last-mile delivery becoming one of the main emission sources. Delivery vehicles operating at the last-mile travel long routes to deliver to customers an array of consignment parcels in varying numbers and weights, and therefore these vehicles play a major role in increasing emissions and air pollutants. The work reported in this paper aims to address these challenges by developing an IoT platform to measure and report on real-world last-mile delivery emissions. Such evaluations help to understand the factors contributing to freight emissions so that appropriate mitigation measures are implemented. Unlike previous research that was completed in controlled laboratory settings, the data collected in this research were from a delivery vehicle under real-world traffic and driving conditions. The IoT platform was tested to provide contextualised reporting by taking into account three main contexts including vehicle, environment and driving behaviours. This approach to data collection enabled the analysis of parcel level emissions and correlation of the vehicle characteristics, road conditions, ambient temperature and other environmental factors and driving behaviour that have an impact on emissions. The raw data collected from the sensors were analysed in real-time in the IoT platform, and the results showed a trade-off between parcel weight and total distance travelled which must be considered when selecting the best delivery order for reducing emissions. Overall, the study demonstrated the feasibility of the IoT platform in collecting the desired levels of data and providing detailed analysis of emissions at the parcel level. This type of micro-level understanding provides an important knowledge base for the enhancement of delivery processes and reduction of last-mile delivery emissions.

## 1. Introduction

Fossil fuel combustion has made vehicles one of the main sources of greenhouse gas (GHG) emissions. In Australia, transport is the second main sources of GHG emissions after electricity, and this emission rate is on the increase. In 2017, the per capita CO_2_ emission produced by transport sector in Austerlia was 32.7 tons. More importantly, road transport in Australia contributes to 85% of transport sector pollution and GHG emissions, which is more than global average [1]. One of the main transport sectors which contributes to GHG emissions is last-mile delivery. In particular, rapid growth of e-commerce has significantly increased the number of last-mile deliveries in the last decade [2,3,4]. Last-mile delivery emissions have been discussed in previous studies [5,6,7,8]. However, research to date has concentrated on estimating the last-mile delivery emissions based on theoretical data about vehicle emission and have not conducted field experiments to determine last-mile delivery emissions.

This paper utilises the Internet of Things (IoT) to deploy multiple sensors on a delivery van to analyse the emissions at parcel level in real-time. The resulting data provide comprehensive information on how different factors can impact delivery and how the delivery process can be enhanced to reduce the last-mile delivery emission.

Vehicle emissions are an inevitable consequence of fossil fuel combustion, but there are certain factors which impact vehicle fuel combustion and emission rates that can be monitored and addressed. These factors can be categorised into several major categories including road condition, driving style, vehicle condition, vehicle mass and weather condition [9].

In fuel consumption analysis research, driving style is categorised into two main categories which are aggressive driving and eco-driving. Aggressive driving refers to high acceleration and deceleration and high speed with sudden breaking patterns. Eco-driving, on the other hand, refers to smooth acceleration and deceleration, optimal gear shifting, and driving with optimal speed. Vehicle condition is another category that impacts vehicle emissions and includes multiple factors such as lubrication, tyres condition, engine tune and air filter. Another aspect impacting vehicle emissions is weather conditions, with rain, snow and ambient temperature effecting fuel consumption [9]. While each of these categories has been a focal point of other research aiming to determine their impact on fuel consumption and vehicle emissions, most of these studies have been conducted in a laboratory environment on chassis dynamometers. Studies show that the results of such tests in controlled laboratory environments are remarkably different from actual vehicles emissions in the real world [10,11,12]. Weiss et al. [10] performed emissions testing in both laboratory and real-world contexts. They argue that, even though that laboratory testing can be used to perform repetitive tests in identical conditions to compare the acquired results, the laboratory environment fails to capture all the factors which impact fuel consumption and vehicle emissions in real-world. Therefore, they argue that there is need for data collection under ordinary operating condition on the road to complement the laboratory data and obtain accurate information to find the correlation between different factors and vehicle emissions.

These studies demonstrate that, in order to obtain comprehensive and accurate data regarding last-mile delivery emissions and their correlation with internal and external factors, there is need to perform field evaluation in real-world context. Although obtaining fuel consumption and vehicle emissions data in a real-world environment would be beneficial to better determine various emission factors impact, but performing such tests has certain challenges. One of the challenges is that laboratory devices are designed to be in fixed positions and are usually connected to the vehicle using wired communication technologies. In addition, gas analyser devices are mostly designed to collect data from cars in stationary mode. These facts hinder deployment of sensors and gas analysers on a moving car. In order to capture live data from vehicles in real-world environment the devices which are used in laboratory must be modified to transfer live data to cloud via wireless communication. Technological advancement and emergence of the IoT has provided substantial advantages to address similar challenges in capturing and processing live and heterogeneous data from multiple sensors in several real-world applications such as precision agriculture, smart cities, healthcare, environmental monitoring and so forth [13,14,15,16,17,18].

The IoT enables the automation of data collection with different types of sensors and integration of various data types into a single data model without human interaction. In addition, through edge computing, the IoT enables primary data processing in the same location where data are collected by sensors. The data are then transferred from edge device to the IoT cloud for real-time visualisation and data analysis. To the best of our knowledge, no field experiment to determine last-mile delivery emission at a parcel level has been conducted before. Such research can provide micro level understanding of last-mile delivery vehicles emission and can provide the information required to improve the efficiency and effectiveness of last-mile delivery procedures.

The main contribution of this research is the design, implementation, and evaluation of an IoT-based emission monitoring platform (referred to as ParcEMon) which enables parcel level emission analysis of last-mile delivery vehicles in real-world contexts.

The rest of this paper is organised as follows: Section 2 discusses the related work, Section 3 presents the methodology including platform architecture, platform implementation and data collection process. Section 4 presents the results of the research. Section 5 concludes this paper.

## 2. Related Work

Different modes of transport such as road, rail, aviation and shipping all result in the emission of GHG and air pollutants through fossil fuel combustion. However, the amount of emission across each transport mode is different with road transport being the most prolific producer of emissions in this sector. Road transport causes around 12% of entire global GHG emissions [19] with light duty vehicles contributing around 72% of this value [20]. Due to the fact that light duty vehicles such as vans are among the main vehicles used in last-mile deliveries, last-mile deliveries must be considered a contributor to GHG emissions. Although emission factors are highly intertwined, research identifies the most prominent emission factors to include driving behaviour, vehicle condition, vehicle mass, aerodynamics, road condition and weather condition.

One of the main factors which impact vehicle emission is driving behaviour. Merkisz et al. [21] conducted a research to measure driving style influence on CO_2_ emission in the real environment. They characterised driving behaviour into three different eco, normal and aggressive styles. Their research shows that eco-driving results in 4.5% less fuel consumption compared to normal driving style and 12.4% less compared to aggressive driving style. Allison et al. [22] argue that eco-driving training has a short-term impact on drivers’ behaviour and after a short period they return to their normal habit of driving. They mention that there is a need for a constant feedback mechanism to continuously inform drivers about the financial and environmental benefits of their eco-driving behaviour. The IoT can play a major role in implementing such real-time feedback systems to continuously encourage the drivers to follow eco-driving behaviour.

Different aspects of vehicle conditions such as lubricants, tyres and engine maintenance can impact fuel consumption. Around 25% of vehicle energy is used to overcome friction in different components of vehicles and using low viscosity lubricants can improve energy consumption. Lowering internal friction in an engine using a suitable motor oil can reduce fuel consumption by 2.5%. Tyres directly impact vehicle resistance which in turn impacts fuel consumption. Low resistance tyres can reduce fuel consumption by 3%. Misaligned wheels and poorly tuned engines are among other vehicle conditions which adversely impact fuel consumption. Flaws like this in vehicles can increase fuel consumption by 3.5% [9]. Vehicle weight is another factor which directly impacts fuel consumption. Zervas et al. [23] argue that, in order to control future gas emission, not only the efficiency of cars in fuel consumption must increase but also their weight must not exceed certain upper limits to reduce fuel consumption. They conducted research on passengers cars and analysed the CO_2_ emission reduction when the weight of cars decreases. The result shows that, when compared to the reference 1600 kg weight limit, cars with 1400 kg weight generate 9% less CO_2_, cars with 1200 kg weight generate 16% less CO_2_ and cars with 1000 kg weight generate 28% less CO_2_.

Studies show that driving uphill can increase fuel consumption by 13%. Road roughness and unevenness also can increase fuel consumption by 2.7% [9]. Zabaar and Chatti [24] conducted research to analyse road roughness on fuel consumption. They used five vehicles in field trials with different weights including a medium car, SUV, van, light truck and heavy truck. They argue that the impact of roughness is intertwined with other factors including vehicle weight, aerodynamics, temperature and road grade. Although several road factors would impact fuel consumption and emission rate, but the most impactful road characteristics on fuel consumption is from traffic congestion, based on the number of vehicles on the road and traffic status. Vehicles on congested roads with heavy traffic condition require repeated decreases and increases in the speed over a long period of time and such fluctuations in speed increase fuel consumption. Greenwood et al. [25] conducted a test to analyse the impact of traffic congestion on fuel consumption and emissions. They found out that fuel consumed when compared to steady speed consumption for a real-life section of motorway increased by around 13% over a 24 h period. Similarly, various vehicle emissions increased by as much as 25%.

Different weather conditions such as precipitation, temperature and air density also impact fuel consumption. Rain and snow both impact the rolling resistance of a car as well as the road surface characteristics. For a depth of one mm, rain can increase fuel consumption by 30%. Temperature is another factor which impacts tyres and also engines due to a cold start. A study shows that temperature between 0 to 20 can increase fuel consumption by 10% [9]. Saboohi and Farzaneh [26] developed a model for eco-driving based on least fuel consumption. They argue that air resistance has the highest impact on fuel consumption compared to other weather-related emission factors. In addition to direct impacts on vehicle and road conditions, various weather conditions can also increase fuel consumption and vehicle emissions by forcing the driver to regularly decelerate and accelerate and prevent eco-driving style.

Laboratory, simulation and real-world context are the environments where data analysis is performed to determine vehicle emissions and air pollutants.

Pelkmans and Debal [11] have performed data collection using a chassis dynamometer to compare laboratory emission with on-road emissions. The data which were captured include speed, relative positive acceleration (RPA), fuel consumption and CO_2_, CO, NOx, THC and PM emissions. Based on their findings, the emissions rate in laboratory is 10–20% less compared to on-road emission. Weiss et al. [10] used a portable emission measurement system (PEMS) to analyse on-road emission of light duty vehicles in Italy. They installed gas analyser and other components including GPS, humidity, temperature and pressure sensors inside the test vehicles and the data were stored locally. The test was conducted using 12 light duty vehicles on four different routes with different characteristics representing rural, urban, uphill/downhill and motorway driving. They argue that their test provides accurate data which indicate on-road NOx emissions of light duty diesel vehicles exceeds Euro 3–5 emission limits, whereas on-road CO and THC emissions generally remain below the Euro 3–5 emission limits.

Miles et al. [27] propose an IoT-based decision support system for monitoring and mitigating pollution in smart cities. They used the IoT capability to integrate data from multiple sources to determine the factors which impact vehicle emission. They integrated vehicle, weather and traffic data using an IoT platform to improve their decision-making process and select appropriate mitigation strategies such as signal optimisation, heavy vehicle ban, parking regulations and road closure or diversion. However, the data collection from vehicle emissions is not IoT-based and standardised vehicle emission models were integrated into the IoT platform to simulate the data.

Much of the prior research conducted to analyse how various emission factors impact fuel consumption and vehicle emissions has acquired data for analysis either from laboratory or from simulation applications. Even though such data can reveal significant information regarding correlation between emission factors and fuel consumption, they cannot fully implement real on-road contexts and characteristics [11]. Therefore, there is always a certain data gap between laboratory data and on-road data. Moreover, to the best of our knowledge, no research has been conducted so far to integrate the IoT capabilities with an advanced industrial gas analyser to analyse vehicle on-road emission in real-time. As a result, the focus of this research is to utilise the IoT capabilities to capture data from multiple emission factors in real on-road contexts in order to analyse parcel level emissions in last-mile delivery processes. The architecture, implementation and data collection of the ParcEMON IoT-based emission monitoring system are addressed in the following section.

## 3. Methodology

**Figure 1:**
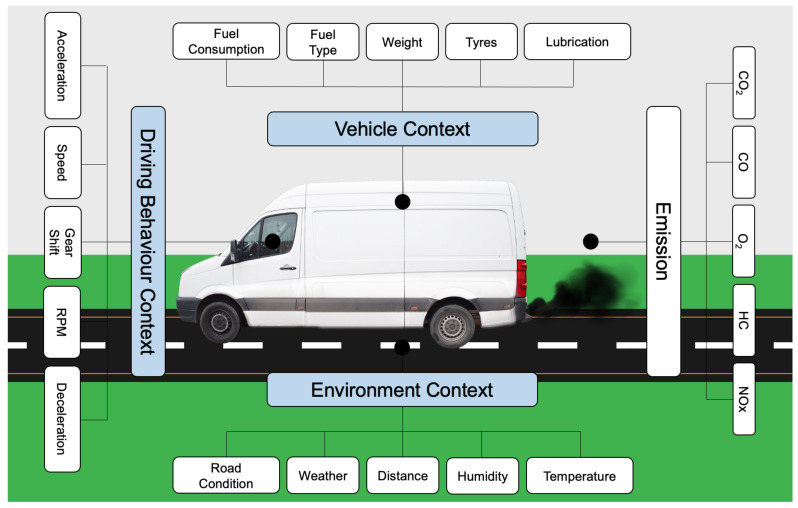
Emission impacting contexts and related parameters.

In this research, contextual data [28] regarding three different areas of impact on emissions are gathered. These contexts include environment, vehicle and driver behaviour contexts. Each of these contexts have certain parameters which can effect the GHG emission rate. Figure 1 shows the impacting contexts and related parameters.

Data collection from the discussed contexts in Figure 1 was conducted using several IoT-based sensors from a van in last-mile deliveries. Data collection was conducted during the autumn season where van emissions were monitored in real time via the proposed ParcEMon platform. The van loaded the goods to be delivered and started the deliveries to different locations from 7:00 a.m. Different data including speed, acceleration, deceleration, gear shift, RPM, external temperature, road condition and vehicle path were captured. The data were then transferred to the IoT cloud using an edge device installed in the van for further data analysis and identification of their correlation with vehicle emissions. In Section 3.1, platform architecture is addressed, and, in Section 3.2, platform implementation is discussed.

### 3.1. Platform Architecture

**Figure 2:**
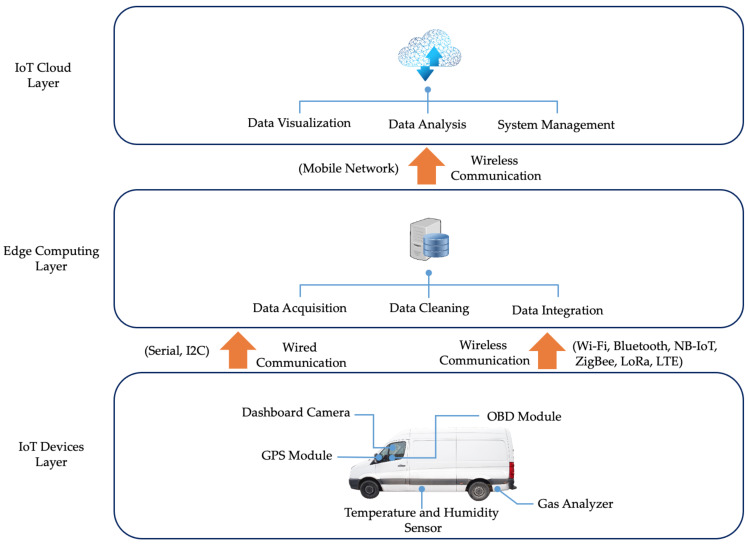
ParcEMon platform architecture.

In this research, an IoT testbed was developed to enable data capturing of a last-mile delivery van in a real-world context. The developed platform is based on a three layer IoT architecture including device, edge and application layers. The data between these layers can be exchanged through a wide range of wired and wireless communication protocols. The IoT architecture and the communication protocols are depicted in Figure 2.

The device layer includes the physical components of the IoT-based emission monitoring platform. This includes the sensors which are used to capture data regarding environment, vehicle and driving behaviour contexts. The data from the device layer were transferred to the edge layer using both wired and wireless communications. In the edge layer, three data processing operations including data acquisition, data cleaning and data integration were conducted using a Raspberry PI single board computer (SBC). Data acquisition refers to the process of sampling the signals transferred from the IoT device and converting them into numeric values to be manipulated by the data analysis software. Data acquisition is followed by data integration where data from multiple sensors with different data types are integrated into a single structured database for further processing. Data cleaning is also performed on the edge layer where incorrect, corrupted and incomplete data are removed from the data set. The data are then transferred to the application layer using a mobile network for data visualisation and data analysis. For data visualisation, an interactive dashboard was designed to present the acquired data from sensors in the IoT device layer. The dashboard also enables the management of the emission monitoring platform by providing an interactive interface for the system administrator. This enables different data sets to be filtered and generate different type of reports with different levels of granularity for data analysis purposes. In addition, the system management component enables additional data, such as the log of parcel size and delivery times, to be added into the database and integrated with the sensor readings from the IoT device layer. A detailed list of sensors, communication technologies and dashboard functionalities are discussed in Section 3.2.

### 3.2. Platform Implementation

Since the gas analyser receives and reads toxic gases form the vehicle emissions, it was necessary to design and install all sensor components outside of the vehicle. The gas analyser and its components were installed on the exterior chassis of the vehicle in such a way as to prevent any object or road bump damage to the devices. The temperature sensor was also installed underneath the van to capture ambient temperature. Other sensors including OBD, dashboard camera, GPS and the edge device were installed inside the driver cabin.

The implemented platform included multiple hardware and software components which are as follows.

- On-Board Diagnostics (OBD) Module: An OBD module was used to read vehicle performance data throughout the deliveries. The data which were captured by OBD included speed, acceleration, deceleration, and gear shift. The data were transferred to the edge device using Bluetooth communication. The OBD module used in this paper supported OBD version 2 with the frequency range of 2402 to 2480 MHz, supply voltage of 12 V, and data transmission range of up to 10 m.
- Dashboard Camera: A dashboard camera was used to record an entire trip in HD quality. The data were used to check the road condition and special events in case of sudden changes in OBD data to better perceive the impact of road condition, special events and driver behaviour on vehicle emission. The dashboard camera data were locally stored on an SD card with time and GPS location integrated in the video. Furthermore, a low resolution video was sent to the dashboard to show the real-time situation of the vehicle.
- GPS Module: A GPS module was integrated into the platform to continuously record the vehicle location. The data were used to determine the vehicle path and distance travelled and also to determine whether vehicle was doing deliveries in rural or urban areas. The GPS data were transferred to the edge device via USB communication. The GPS module used in this paper was GlobalSat BU-353-S4 utilising SiRF STAR IV GSD4e GPS chipset.
- Temperature and Humidity Sensor: A temperature sensor was installed on an Arduino microcontroller to continuously record the ambient temperature. The sensor was deployed underneath the van on the opposite side of the exhaust to prevent exhaust heat from impacting the sensor readings. The data were then transferred to the edge device using USB communication. The ambient temperature was used to analyse the impact of temperature on fuel consumption and vehicle emission in particular the impact of early morning cold weather on engine performance. The ambient condition sensor used in this paper was an SHT31 weather-proof temperature and humidity sensor.
- Gas Analyser: The vehicle gas emission (i.e., CO_2_) was measured using an industrial gas analyser. The gas analyser used in this paper was able to capture five gases including CO, CO_2_, O_2_, HC and NOx. However, in this paper, we only report CO_2_, which is the most prolific GHG. The gas analyser included a nozzle which was fixed inside the exhaust and transferred the exhaust emissions to the gas analyser module using a pipe. The data were then transferred from a gas analyser to the edge device using serial communication. The specification of the gas analyser used in this paper is shown in Table 1.
- Edge device: The edge device was used to collect data from all the sensors and transferred the data to the IoT cloud. The edge device was designed using a Raspberry Pi single board computer. The data were captured using different communication protocols including USB, serial and Bluetooth based on each sensor specification. The data were transferred to the IoT cloud using Wi-Fi communication. In addition, the edge device was equipped with a 60,000 mAh battery, which was the power source of all sensors except the OBD which is directly powered via the vehicle OBD port. At the end of the experiment, 70% of the battery power was used by the deployed components. The components of the edge device are described in Table 2.

**Table 1:** Gas analyser specifications.

Gases Measured	CO (Carbon Monoxide) HC (HydroCarbons—Hexane (Gasoline) Propane (LPG), or Methane (CNG or LNG) CO_2_ (Carbon Dioxide) O_2_ (Oxygen) NO (NOx, Nitric Oxide)
Analysis Method	CO, HC, CO_2_: NDIR (Non Dispersive Infra-Red) O_2_, NO: Electro-Chemical Sensor
Reporting Ranges	CO: 0–10.00% HC (Hexane and Propane): 0–9999 ppm HC (Methane) 0.000–5.000% CO_2_: 0–20.0% O_2_: 0.0–25.0% NO: 0–5000 ppm
Resolution	CO: 0.01% HC (Hexane, Propane): 1 ppm HC (Methane): 0.001% CO_2_: 0.1% O_2_: 0.1% NO: 1 ppm
Accuracy	All gas channels +/−5% relative to gas reading
Repeatability	All gas channels +/−3% full scale
Response Time	Less than 8 s to 90% final value.
Warm-Up Time	30 s to 10% accuracy 5 min to full accuracy. (Constant ambient conditions)
Gas Sample Rate	350 mL/min typical. (Flow control pneumatics system).

**Table 2:** Edge device components.

Components	Description
Raspberry Pi 4	Broadcom BCM2711, Quad core Cortex-A72 (ARM v8) 64-bit SoC @ 1.5 GHz, 4 GB LPDDR4-3200 SDRAM 2.4 GHz and 5.0 GHz IEEE 802.11 ac wireless, Bluetooth 5.0, BLE, Gigabit Ethernet Raspberry Pi standard 40 pin GPIO header Micro-SD card slot for loading operating system and data storage 5V DC via USB-C connector
Power Bank	Battery Type: Li-Polymer Capacity: 64,000 mAh DC Input: 19 V 2 AAC Output: 220 V 50 HZ 130 W TYPE-C Output: 5 V/9 V/12 V 3 A USB Output: 5 V/9 V/12 V 3 A Recharging Time: 8–10 h
7 inches Touchscreen Display	TFT Display Screen Dimensions: 194 mm × 110 mm × 20 mm Viewable screen size: 155 mm × 86 mm Screen Resolution 800 × 480 pixels 10 finger capacitive touch Connects to the Raspberry Pi board using a ribbon cable connected to the DSI port.
Huawei 4G USB E3372	FDD: DD800/900/1800/2100/2600 UMTS: 900/2100 GSM: 850/900/1800/1900 LTE FDD: Cat4 DL: 150 Mbps/UL: 50 Mbps @20 M BW UMTS: DCHSPA+: 42/5.76 Mbps; 21 M/5.76 Mbps; 14 M/5.76 M HSUPA: 7.2 M/5.76 M 2G: EDGE packet data service of up to 236.8 kbps

**Figure 3:**
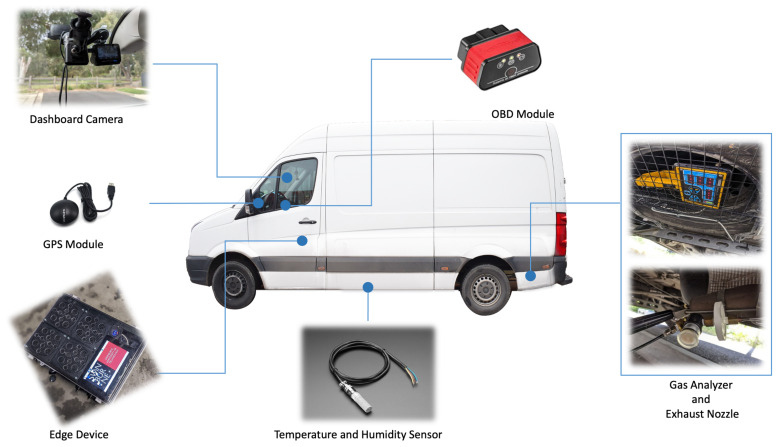
ParcEMon platform implementation.

Figure 3 illustrates the platform implementation and the IoT components.

The acquired data from the sensors were transferred to the IoT cloud platform for real-time visualisation and data analysis.

**Figure 4:**
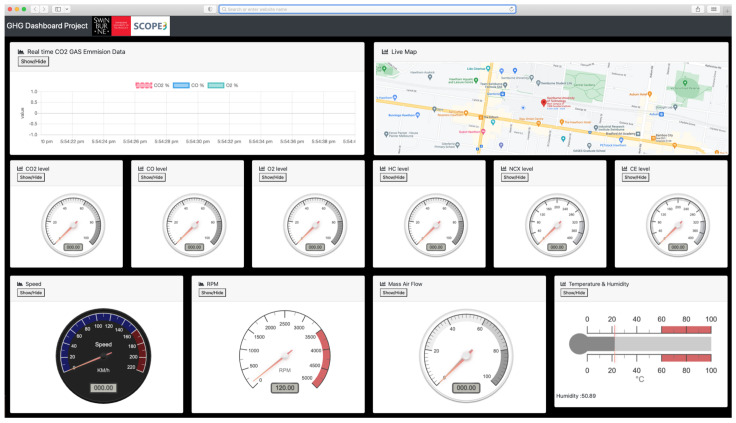
ParcEMon online dashboard.

The dashboard presented in Figure 4 is a web-based online application that enables monitoring of the ParcEMon in real-time to ensure the platform, and its components are operational during the field-trial. In addition, it reports and records the vehicle location and status. The dashboard also generates several emission reports from the collected data with a preferred granularity level. Furthermore, this dashboard can be extended to facilitate lower emission route suggestion based on the historical and real-time data.

### 3.3. Data Collection

**Figure 5:**
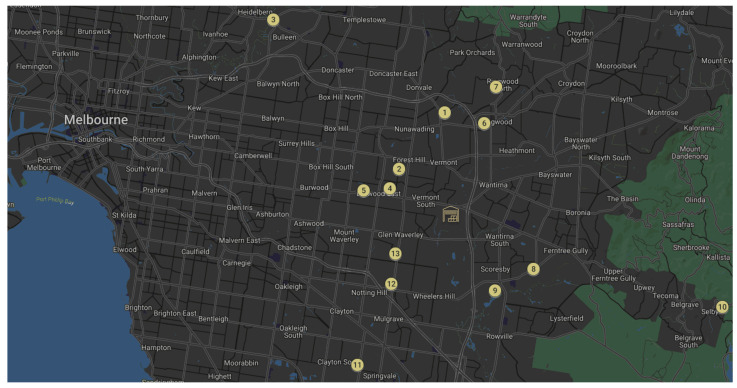
The warehouse location and the order of 13 last mile parcel deliveries.

In this research, the IoT sensors, including a gas analyser, were deployed to capture live data from a delivery van. The van was working for an Australian household chain in the city of Melbourne and was handling last-mile deliveries. The acquired data covered a wide range of parameters which can be divided into two categories. The first category relates to environmental parameters including weather condition, temperature, humidity and road condition. The second category relates to car parameters including engine’s revolution per minute (RPM), acceleration, deceleration, gear shift, Global Positioning System (GPS) coordinates, distance travelled and the travel path. The data from the gas analyser and other sensors were transferred to an edge device installed in the van via different communication protocols and subsequently the integrated data were transferred to the IoT cloud. The last-mile delivery vehicle emission and the data related to other contexts described in Section 3 were recorded from the vehicle departure point from the warehouse until the last parcel delivery. As it is shown in Figure 5, the warehouse is represented using the warehouse icon and each parcel, and its order of delivery is represented in circle icons. In total, data for 13 delivery points were recorded. The delivery points were in different suburbs with different road congestion and road conditions.

**Figure 6:**
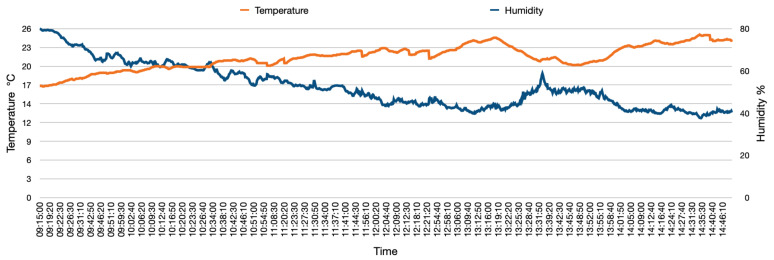
Ambient temperature and humidity during the field-trial.

The data collection was captured during the autumn season with a temperature range between around 17 °C to 26 °C. The ambient temperature and humidity were recorded to ensure that the range of these two factors is within the operational range of the gas analyser sensor. Figure 6 shows the ambient temperature and sensor thorough the delivery process.

**Figure 7:**
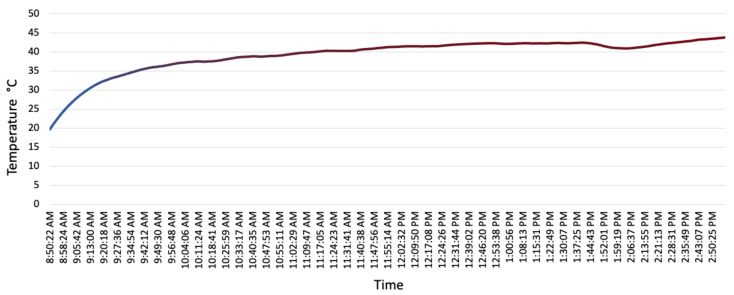
Gas analyser operating temperature during the field-trial.

The gas analyser temperature shows a sudden increase at the beginning of the delivery process. However, after an hour, the gas analyser temperature increase rate reduces and shows a mild increase around 5 °C for the rest of the delivery day. This gas sensor temperature increase is due to exhaust gas heat generated throughout the delivery process. Figure 7 shows the gas analyser sensor temperature throughout the delivery process.

For each parcel, the weight and the time of delivery were recorded. In addition, the consignment type for each parcel, which represents the size category of the parcel, is registered. The consignment types include economy, small and medium parcels, which represent the parcel weight from light to heavy. The lightest parcel was parcel No. 6 with a weight of 1 kg, and the heaviest parcel was parcel No. 1 with the weight of 194 kg. Other information including the delivery address, suburb and the postcode are also recorded to identify the suburb changes during the last-mile deliveries. The distance from each delivery point to the next delivery point is also recorded. These data can be used to analyse how many emissions have been released for each parcel based on the distance travelled, and this can be beneficial for analysing the best possible path to reduce the emission for all deliveries in a single day. Table 3 shows the recorded data for last-mile delivery on a single day which includes 13 deliveries.

## 4. Results

**Figure 8:**
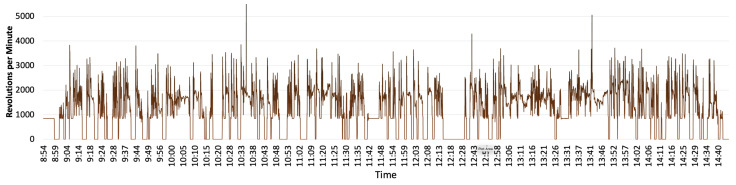
Engine’s RPM data collected during the field-trial.

The acquired data of this research are analysed from four different perspectives to determine the factors which impact each parcel delivery’s CO_2_ emission. These perspectives include van weight at each delivery point, distance travelled, interruption and driver behaviour. The driver behaviour is determined by analysing the vehicle Revolution per Minute (RPM), speed, acceleration and deceleration data. These factors impact the fuel consumption and subsequently the CO_2_ emission. Figure 8 shows the RPM fluctuation during the last-mile deliveries. The deliveries which have resulted in higher RPM than average can contribute to more fuel consumption. The data also enable determining the road condition such as road gradient or traffic congestion.

**Figure 9:**
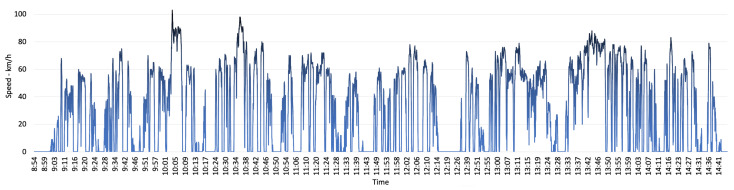
Vehicle’s speed data collected during the field-trial.

Similarly, the speed chart represented in Figure 9 enables analysis of driver behaviour in different conditions. In Victoria, the default speed limit for built-up areas is 50 km/h and on the highway it is 100 km/h. Using the speed chart, it can be determined in which time of the day the delivery van has been on the highway or in built-up areas. For example, the data points on 10:11 and 10:40 show that the vehicle has been on the highway to deliver parcel No. 3. This can be used to analyse the impact of constant speed on parcel level emission in comparison to the condition where there has been constant halts in vehicle movement such as traffic condition in built-up areas. This can provide beneficial data to determine the best possible route to reduce the deliveries emission.

**Figure 10:**
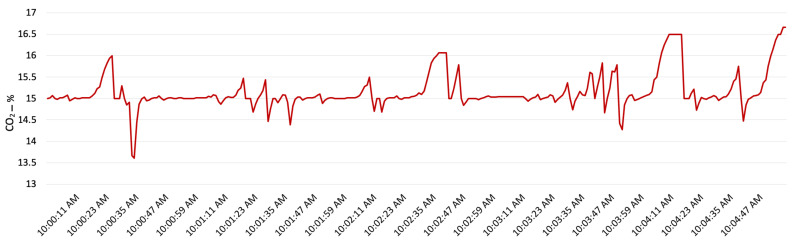
CO_2_ percentage in emitted gas from the tailpipe.

**Figure 11:**
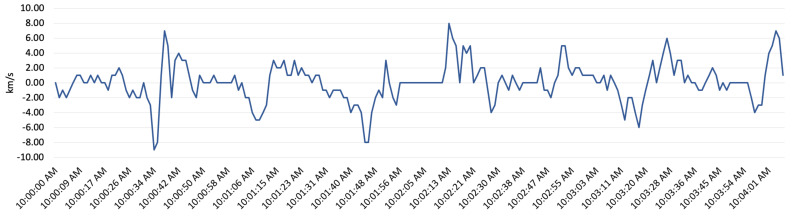
Acceleration and deceleration of the vehicle.

The other factors which reflect driver behaviour are acceleration and deceleration. In order to show the impact of acceleration and deceleration on CO_2_ emission, a five minute segment of driving and the CO_2_ emission has been shown in Figure 10 and Figure 11. Figure 11 shows the acceleration and deceleration, and Figure 10 shows the CO_2_ emission percentage which results a few seconds after acceleration and deceleration. Figure 11 shows that the acceleration and deceleration can slightly impact the CO_2_ percentage in exhaust gas; however, most of the time the CO_2_ percentage is 15%. This value is an ideal fuel combustion level for a diesel engine vehicle such as the delivery van engine examined in this research.

**Figure 12:**
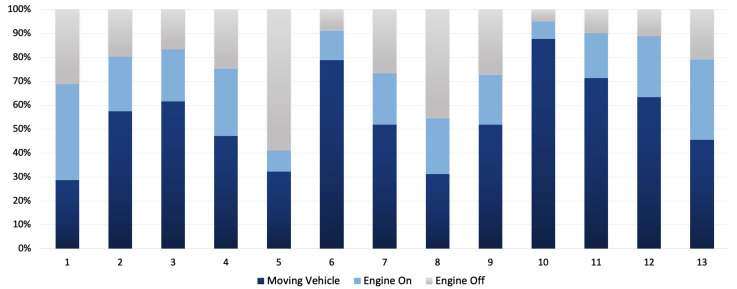
Engine status and vehicle movement (i.e., speed greater than zero) during each delivery in the field-trial.

Another factor which must also be considered is interruptions during the deliveries. The interruptions during delivery such as a driver’s stop for breakfast and lunch have been removed from the data to correctly acquire the emission ratio compared to duration for each parcel delivery. Figure 12 shows the duration for each delivery when the van has been on, and also the duration when the van has been moving (having speed more than 0 km) in comparison to the engine running time.

As it can be seen in Figure 12, parcel No. 5 has the highest engine off duration which indicates an interruption in the delivery. Parcel No. 10 has the highest engine on duration and also the vehicle movement in that period. These data can be used to show how long the delivery van has been utilised in each parcel delivery. In addition to driver behaviour and interruptions, parcel weight and the distance travelled also impact the emission level for each parcel delivery. The parcel weight has been addressed in Table 3. Table 4 integrates distance and interruption factors along with previously discussed driver behaviour for each parcel.

Each row presents the data from the previous delivery point starting from warehouse to delivery 1 in row No. 1. In total, 143 km has been travelled by the last-mile delivery van. Parcel 3 delivery has the highest maximum speed which shows the driver has been driving on the highway. This parcel has taken 30 min to be delivered over a 16 km distance. The longest path travelled for a parcel belongs to parcel No. 11 where 32 km has been travelled to make the parcel delivery.

**Figure 13:**
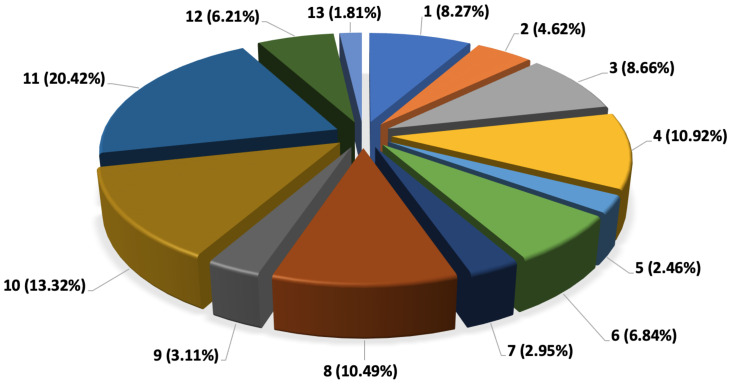
CO_2_ emission percentage per delivery.

The CO_2_ emission for each parcel has been calculated based on the parcel contribution percentage to overall CO_2_ emission. These data are presented in Figure 13.

As it can be noticed from Figure 13, parcel No. 13 with a weight of 30 kg and a distance of 2 km have the lowest emission of 1.81% from overall emission. On the other hand, parcel No. 11 with weight of 50 kg and the distance of 32 km has the highest CO_2_ emission percentage. This parcel has contributed to 20.42% of overall CO_2_ emission during the delivery day. However, it must be also be taken into consideration that the weight of parcels which have not yet been delivered including parcels 12 and 13 can contribute to this number as they are still being carried by the delivery van during parcel 11 delivery. As a result, the cumulative weight of the van through entire delivery has been taken into consideration to determine the CO_2_ emission of each parcel by considering the weight of other existing parcels in the van at each delivery point. Figure 13 shows the cumulative weight of the van at each delivery point. The weight of the van was 2000 kg, which is also added to the overall parcel weight to have the cumulative weight at each delivery point.

**Figure 14:**
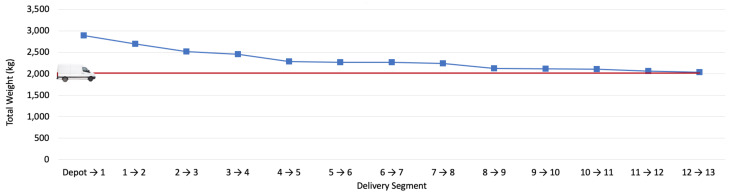
Van and parcels weight during the field-trial.

Figure 14 shows the emission of each parcel based on the cumulative weight of the vehicle.

**Figure 15:**
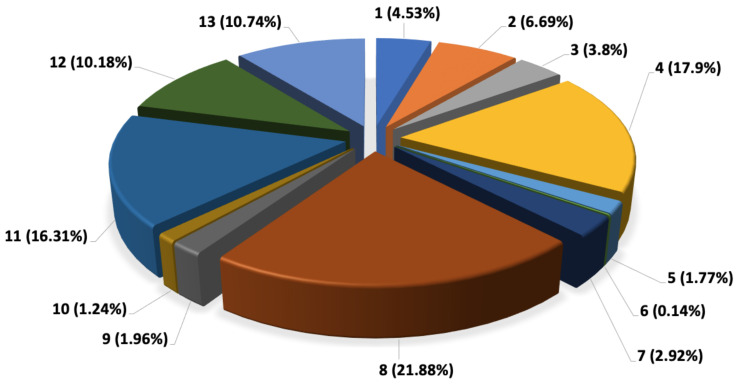
CO_2_ cumulative emission percentage per consignment.

As shown in Figure 15 after considering the cumulative weight of the van, the parcels’ contribution to overall CO_2_ changes considerably. Parcel No. 8 with weight of 199 kg contributes to 21.88% of overall CO_2_ emission. This parcel has been in the van for 76 km before it is delivered. Considering the fact that this parcel is a heavy weight parcel, it has resulted in high CO_2_ emission over a long distance being on the move by delivery van. Parcel No. 11, which showed the highest CO_2_ emission percentage when considered individually, after cumulative weight analysis is dropped to the third highest contributing parcel after parcel No. 8 and No. 4. In cumulative analysis, parcel No.11 has contributed to 16.31% of CO_2_ contribution. Parcel No. 11 has been in the van for a distance of 133 km, but since its weight is 50 kg, which is much less than parcel No. 8, it therefore has continued to be less than CO_2_ percentage compared to parcel No. 8. Parcel No. 4, despite the fact that it has only been in the van for 45 km, due to its heavy weight of 176 kg has resulted in 17.9% of overall CO_2_ emission. Parcel No. 13, which had only 1.81% contribution to CO_2_ emission before cumulative weight analysis, shows 10.74% contribution to overall CO_2_ emission due to being in the vehicle until the end of the delivery day.

The results of this research show that the cumulative weight and the distance travelled can remarkably impact the parcel level CO_2_ emission. Delivering heavy weight parcels at the beginning of delivery day can reduce the CO_2_ emission reporting. However, it must also be considered that delivering the heavy weight parcels at the beginning of delivery day can increase the total path travelled by the delivery van. Therefore, although the CO_2_ emission from a weight perspective could be reduced, the CO_2_ emission from a distance travelled perspective would increase. This shows that there is a trade-off between parcel weight and total distance which must be considered when selecting the best delivery order for reducing CO_2_ emission as much as possible.

During this research, several limitations were identified which impacted the platform implementation and data analysis process. One of the limitations was that the gas analyser functionality could be impacted by environmental factors such as road bumps, mud and water splash. In addition, the gas analyser could not be located in a sealed enclosure since such enclosure prevents air flow and impacts the gas analyser reading. Moreover, the gas analyser performs periodic self calibration which results in a few seconds of data loss after each calibration cycle. Another limitation was that the weight of the driver and other possible passengers and materials inside the van other than the parcels were not considered in this research findings.

Future research directions in this topic should consider instrumentation and measurement of a large number of delivery vehicles representing the Australian vehicle fleet. The trial should also be for longer duration and cover different ambient conditions to capture the range of representative real-life conditions that are typical for Australian parcel delivery operations and road network conditions.

## 5. Conclusions

The transition to a low carbon and low emissions future depends on mitigating climate change in all sectors of the economy. Within the transport sector, freight transport networks, supply chains and last-mile deliveries present particular challenges to successful emissions mitigation. This paper presented the design, implementation and evaluation of an IoT platform for real-time parcel level last-mile delivery emissions reporting. The study demonstrated the feasibility of the technology, which comprised multiple IoT sensors, in measuring real-time emissions per parcel in real-world last-mile delivery vehicles. The results showed that the cumulative vehicle weight and the total distance travelled have substantial impacts on parcel level emissions. The findings present an approach for moving away from static models of carbon emissions assessment, to a more detailed analysis of on road data, including driving conditions and carbon intensity measures across the last mile in the logistics chain. These findings provide freight delivery operators with valuable insights in optimising their delivery schedules such that heavy-weight parcels are delivered at the start of the vehicle’s journey to minimise the total distance travelled with heavy loads, which would result in substantial reduction of the vehicle’s carbon footprint during those deliveries. However, the results also showed that there is a trade-off between delivery of heavy items first, and the total distance travelled by the vehicle during the day, in situations where the heavy parcels are located further away from the distribution centres. Data from the IoT platform developed in this study can help in ameliorating the negative impacts of such trade-offs by providing micro-level data that can be used to determine optimal locations for distributions’ centres around the city, which would result in reducing the distances travelled and subsequently the levels of emissions emitted by each delivery vehicle.

## Figures and Tables

**Table 3:** Consignment delivered during the field-trial.

No	Consignment Type	Weight (kg)	Delivery Time	Approximate Delivery Street	Delivery Suburb	Delivery Postcode
1	Medium	194	09:37	Rye Street	Mitcham	3132
2	Medium	179	09:55	Mahoneys Road	Forest Hill	3131
3	Medium	58	10:23	Manningham Road	Bulleen	3105
4	Medium	176	11:01	Ballantyne Street	Burwood East	3151
5	Small	16	11:18	Burwood Highway	Burwood East	3151
6	Economy Parcel	1	11:36	Ringwood Square Shopping Centre	Ringwood	3134
7	Economy Parcel	20	11:48	Lockhart Road	Ringwood North	3134
8	Medium	119	12:27	Ferntree Gully	Knoxfield	3180
9	Economy Parcel	10	13:02	Koornang Road	Scoresby	3179
10	Small	5	13:33	Caroline Street	Selby	3159
11	Medium	50	14:21	Fairbank Road	Clayton	3168
12	Small	29	14:39	Jarrah Court	Glen Waverley	3150
13	Small	30	14:48	Lincoln Avenue	Glen Waverley	3150

**Table 4:** Delivery segments during the field-trial.

Segment	Round per Min		Speed (km/h)		Start	End	Duration hh:mm	Max Acceleration km/s	Max Deceleration km/s	Distance km	Interruption (Engine Off)
	Avg	Max	Avg	Max							
Depot → 1	1064	3821	13	68	8:54	9:40	00:46	11	15	7	31%
1 → 2	1297	3784	26	75	9:40	9:56	00:16	12	20	6	20%
2 → 3	1267	3486	38	103	9:56	10:26	00:30	11	11	16	17%
3 → 4	1188	5824	33	98	10:26	11:05	00:39	13	15	16	25%
4 → 5	1515	3424	28	70	11:06	11:20	00:14	10	10	2	59%
5 → 6	1663	3682	42	72	11:20	11:38	00:18	12	9	11	9%
6 → 7	1310	3085	24	58	11:38	11:49	00:11	8	8	4	27%
7 → 8	1172	3642	26	78	11:49	12:50	01:01	20	15	14	45%
8 → 9	1243	4288	23	73	12:50	1:05	00:15	9	14	4	27%
9 → 10	1624	3673	46	79	13:05	13:33	00:28	13	16	21	5%
10 → 11	1544	5056	43	88	13:33	14:23	00:50	15	15	32	10%
11 → 12	1436	3476	31	83	14:23	14:40	00:17	12	11	8	11%
12 → 13	993	3280	19	79	14:40	14:49	00:09	12	10	2	21%

## References

[1] Stock P., Steffen W., Bourne G., Brailsford L. (2018). Waiting for the Green Light: Transport Solutions to Climate Change.

[2] Siikavirta H., Punakivi M., Kärkkäinen M., Linnanen L. (2002). Effects of e-commerce on greenhouse gas emissions: A case study of grocery home delivery in Finland. J. Ind. Ecol..

[3] Laghaei J., Faghri A., Li M. (2016). Impacts of home shopping on vehicle operations and greenhouse gas emissions: Multi-year regional study. Int. J. Sustain. Dev. World Ecol..

[4] Jiang L., Chang H., Zhao S., Dong J., Lu W. (2019). A travelling salesman problem with carbon emission reduction in the last mile delivery. IEEE Access.

[5] Brown J.R., Guiffrida A.L. (2014). Carbon emissions comparison of last mile delivery versus customer pickup. Int. J. Logist. Res. Appl..

[6] Song L., Guan W., Cherrett T., Li B. (2013). Quantifying the greenhouse gas emissions of local collection-and-delivery points for last-mile deliveries. Transp. Res. Rec..

[7] Wygonik E., Goodchild A.V. (2018). Urban form and last-mile goods movement: Factors affecting vehicle miles travelled and emissions. Transp. Res. Part D Transp. Environ..

[8] Li L., He X., Keoleian G.A., Kim H.C., De Kleine R., Wallington T.J., Kemp N.J. (2021). Life cycle greenhouse gas emissions for last-mile parcel delivery by automated vehicles and robots. Environ. Sci. Technol..

[9] Zacharof N., Fontaras G., Ciuffo B., Tsiakmakis S., Anagnostopoulos K., Marotta A., Pavlovic J. (2016). Review of in Use Factors Affecting the Fuel Consumption and CO_2_ Emissions of Passenger Cars.

[10] Weiss M., Bonnel P., Hummel R., Manfredi U., Colombo R., Lanappe G., Le Lijour P., Sculati M. (2011). Analyzing On-Road Emissions of Light-Duty Vehicles with Portable Emission Measurement Systems (PEMS).

[11] Pelkmans L., Debal P. (2006). Comparison of on-road emissions with emissions measured on chassis dynamometer test cycles. Transp. Res. Part D Transp. Environ..

[12] Ježek I., Drinovec L., Ferrero L., Carriero M., Močnik G. (2015). Determination of car on-road black carbon and particle number emission factors and comparison between mobile and stationary measurements. Atmos. Meas. Tech..

[13] Bagha H., Yavari A., Georgakopoulos D. (2022). Hybrid Sensing Platform for IoT-Based Precision Agriculture. Future Internet.

[14] Yavari A., Jayaraman P.P., Georgakopoulos D., Nepal S. ConTaaS: An approach to internet-scale contextualisation for developing efficient internet of things applications. Proceedings of the 50th Hawaii International Conference on System Sciences.

[15] Gaire R., Sriharsha C., Puthal D., Wijaya H., Kim J., Keshari P., Ranjan R., Buyya R., Ghosh R.K., Shyamasundar R. (2020). Internet of Things (IoT) and cloud computing enabled disaster management. Handbook of Integration of Cloud Computing, Cyber Physical Systems and Internet of Things.

[16] Yavari A., Georgakopoulos D., Stoddart P.R., Shafiei M. Internet of Things-based hydrocarbon sensing for real-time environmental monitoring. Proceedings of the 2019 IEEE 5th World Forum on Internet of Things (WF-IoT).

[17] Forkan A.R.M., Montori F., Georgakopoulos D., Jayaraman P.P., Yavari A., Morshed A. An industrial IoT solution for evaluating workers’ performance via activity recognition. Proceedings of the 2019 IEEE 39th International Conference on Distributed Computing Systems (ICDCS).

[18] Yavari A., Georgakopoulos D., Agrawal H., Korala H., Jayaraman P.P., Milovac J.K. Internet of Things milk spectrum profiling for industry 4.0 dairy and milk manufacturing. Proceedings of the 2020 International Conference on Information Networking (ICOIN).

[19] Ritchie H., Roser M., Rosado P. (2020). CO_2_ and Greenhouse Gas Emissions.

[20] (2022). CO_2_ Emissions from Cars: Facts and Figures (Infographics). European Parliament.

[21] Merkisz J., Andrzejewski M., Merkisz-Guranowska A., Jacyna-Gołda I. (2014). The influence of the driving style on the CO_2_ emissions from a passenger car. J. KONES.

[22] Allison C.K., Stanton N.A. (2019). Eco-driving: The role of feedback in reducing emissions from everyday driving behaviours. Theor. Issues Ergon. Sci..

[23] Zervas E., Lazarou C. (2008). Influence of European passenger cars weight to exhaust CO_2_ emissions. Energy Policy.

[24] Zaabar I., Chatti K. (2010). Calibration of HDM-4 models for estimating the effect of pavement roughness on fuel consumption for US conditions. Transp. Res. Rec..

[25] Greenwood I., Dunn R., Raine R. (2007). Estimating the effects of traffic congestion on fuel consumption and vehicle emissions based on acceleration noise. J. Transp. Eng..

[26] Saboohi Y., Farzaneh H. (2009). Model for developing an eco-driving strategy of a passenger vehicle based on the least fuel consumption. Appl. Energy.

[27] Miles A., Zaslavsky A., Browne C. (2018). IoT-based decision support system for monitoring and mitigating atmospheric pollution in smart cities. J. Decis. Syst..

[28] Yavari A. (2019). Internet of Things Data Contextualisation for Scalable Information Processing, Security, and Privacy. Ph.D. Thesis.

